# Factor Structure of an ICF-Based Measure of Activity and Participations for Adults in Taiwan's Disability Eligibility Determination System

**DOI:** 10.3389/fresc.2022.879898

**Published:** 2022-05-13

**Authors:** Hua-Fang Liao, Chia-Feng Yen, Tzu-Ying Chiu, Wen-Chou Chi, Tsan-Hon Liou, Ben-Sheng Chang, Ting-Fang Wu, Shu-Jen Lu

**Affiliations:** ^1^School and Graduate Institute of Physical Therapy, National Taiwan University, Taipei, Taiwan; ^2^Taiwan Society of ICF, Taipei, Taiwan; ^3^Department of Public Health, Tzu Chi University, Hualien, Taiwan; ^4^Department of Health and Welfare, College of City Management, University of Taipei, Taipei, Taiwan; ^5^Department of Occupational Therapy, Chungshan Medical University, Taichung, Taiwan; ^6^Department of Physical Medicine and Rehabilitation, Shuang Ho Hospital, Taipei Medical University, Taipei, Taiwan; ^7^Department of Physical Medicine and Rehabilitation, School of Medicine, College of Medicine, Taipei Medical University, Taipei, Taiwan; ^8^Department of Psychology, Soochow University, Taipei, Taiwan; ^9^Graduate Institute of Rehabilitation Counseling, National Taiwan Normal University, Taipei, Taiwan; ^10^School of Occupational Therapy, College of Medicine, National Taiwan University, Taipei, Taiwan

**Keywords:** disability evaluation, functioning, ICF, measurement, participation

## Abstract

To assess activity and participation for adults in Taiwan's Disability Eligibility Determination System (DEDS), we developed a measure, the Functioning Disability Evaluation Scale—Adult version (FUNDES-Adult), based on the 36-item interviewer-administered version of the WHO Disability Assessment Schedule 2.0. The purpose of this study was to examine the factor structures of performance and capability dimensions of the FUNDES-Adult. This study followed a methodology research design to investigate the construct validity of the two dimensions of the FUNDES-Adult. Two samples were randomly stratified from the databank of adults with disabilities to examine structural validity by the exploratory factor analysis (EFA) (*n* = 8,730, mean age of 52.9 ± 16.81) and the confirmatory factor analysis (CFA) (*n* = 500, mean age of 54.3 ± 16.81). The results demonstrated that the EFA yielded 5-factor structures for both performance dimension (73.5% variance explained) and capability dimension (75.9% variance explained). The CFA indicated that the second-order factor structures of both dimensions were more parsimonious with adequate fit indices (GFI, NFI, CFI, and TLI ≥ 0.95, RMSEA < 0.09). The results of this study provide evidence that the FUNDES-Adult has acceptable structural validity for use in Taiwan's DEDS. Utility of the FUNDES-Adult in rehabilitation, employment, welfare, and long-term care services needs further study.

## Introduction

According to Taiwan's People with Disabilities Rights Protection Act promulgated in 2007 (1), the local government in Taiwan should issue the disability identification and provide welfare and services based on the framework of the International Classification of Functioning, Disability, and Health (ICF) (2, 3). Taiwan is one of the pioneer countries to use the ICF Chapter code as a basis for the classification of disability and for the application of ICF in the disability eligibility determination system (DEDS) (4–7). In Article 5 of the People with Disabilities Rights Protection Act, people with disabilities refer to those who, with the following deviation or loss resulting from physical or mental impairments, are limited or restricted to be engaged in the ordinary living activities and participation (AP) in the society; and they, after processes of evaluation and assessment by the committee composed of professionals from medicine, social work, special education, and employment counseling and evaluation, can be regarded as suffering one of the following malfunction categories and issued a disability identification. The eight disability types are listed as follows: (1) Mental functions and structures of the nervous system; (2) Sensory functions and pain, the eye, the ear, and the related structures; (3) Functions and structures of/involved in voice and speech; (4) Functions and structures of/related to the cardiovascular, hematological, immunological, and respiratory systems; (5) Functions and structures of/related to the digestive, metabolic, and endocrine systems; (6) Functions and structures of/related to the genitourinary and reproductive systems; (7) Neuromusculoskeletal and movement related functions and structures; (8) Functions and related structures of the skin. The eight disability types or malfunctional categories corresponding to the eight chapters of body functions and structures of the ICF. The statement of the Article 1 of that act also emphasizes the focuses of societal participation in Taiwan government services. The societal participation therefore is one major outcome of welfare and services for people with disabilities. Besides, the ICF proposes that disability, or participation restriction, is the results of interaction among disease, body function and structures, environmental factors, and personal factors (2). In Taiwan's past disability evaluation system, the disability identification was issued after physician diagnosis and impairment examinations only. In the current DEDS system, the disability identification will be issued after a more comprehensive evaluation of body function, AP, and environmental/personal factors and needs assessment by a team (4–7). To assess the status of AP for adult applicants, the World Health Organization Disability Assessment Schedule 2.0 (WHODAS 2.0) (8) was then adapted by the ICF team.

After 5 years' preparation, the ICF-based DEDS have launched nationwide since July 2012, and the disability identification is issued based on the results of the ICF-based disability evaluation by a medical team from the authorized hospitals and on the results of the needs assessment from the local social welfare department (4, 5). The content of the disability evaluation includes tests related to body function and structure codes as well as AP components of the ICF. To assess the status of AP in the ICF-based DEDS, the ICF taskforce group have developed the Functioning Disability Evaluation Scale (FUNDES) since 2007 in Taiwan (5–7). The FUNDES has adult version (FUNDES-Adult) and child version (FUNDES-Child). The FUNDES-Adult has been developed with bilingual translation based on the 36-item interviewer-administered version of the WHODAS 2.0 (8), the mobility trial version for functional assessment (9), and environmental ICF codes (10) that designed and revised at the evaluation tool development phase for the DEDS (6). The FUNDES-Child has been developed based on the Child and Family Follow-up Survey (CFFS) (11).

To take into account, the localization and instrument validity of nationwide application, the changes and revisions of the FUNDES has been on-going based on the feedback of the field testers and experts or from the data analyses nearly every year. Several versions of the FUNDES have been developed (6, 12). Some psychometric properties of the previous FUNDES-Adult, version 5 (13, 14) and FUNDES-Child version (11, 15–17) have been examined and published. The previous construct validity of the FUNDES-Adult version only included the items of 36 items which are the same as WHODAS 2.0 and to follow the same 6-domain structures (13, 18). However, during the revision process, the measurement items have been added for collecting more AP information for people with disability and we did not re-check the psychometric properties of all items in the FUNDES-Adult yet. With the changes in the purpose and background of the assessment tools used, the factor structure of the inspection tools is very much necessary (19), which can assist us in the understanding and analyzing intervention impact of the disability.

This study aimed to examine the factor structures of AP part of the seventh version of the FUNDES-Adult (FUNDES-Adult, version 7) which was revised base on the Taiwan culture and for the purposes of the disability eligibility determination. The FUNDES-Adult, version 7 has applied to the DEDS for a long time, and it is also the basis and reference basis for all future versions and with large number of accumulated cases. Although we also published and used the eighth version later, this version differs only in typography. In the seventh version, we put all the information in one book, including ICF introduction, detailed description of the content and psychometric properties of the FUNDES-Adult and FUNDES-Child, test items, evaluation forms, and flash cards. In the eighth version, there are one FUNDES Manual and one FUNDES Item Booklet. The streamlined FUNDES Item Booklet was designed to assist the testers to evaluating the applicants in the field directly, which made it was not necessary to take the whole heavy FUNDES manual. All questions and measurement procedures of the FUNDES-Adult, version 7, were the same as that of the eighth version, so we have chosen the FUNDES-Adult, version 7, as the target of this study.

Each item of the Domain 1 to 6 of the FUNDES-Adult, version 7, has two dimensions—performance and capability—to measure AP in daily life over the previous 30 days (13, 14, 18). The performance refers to the extent of restriction on participation in daily life and the qualifier of performance is described as what an individual does in his or her current environment. Since the current environment always includes the overall societal context, performance can also be understood as “involvement in a life situation” or “the lived experience” of people in their actual context. As the assistive devices and other persons' assistance changed the performance difficulty level of persons with disabilities (20, 21), for the disability eligibility determination, the FUNDES team decided to add items with capability dimension in the FUNDES-Adult (5). The capability refers to the extent of restriction on participation in a real environment without assisting by any assistive device or persons. The capability dimension therefore captures the extent of difficulty in daily life without environmental supports (13). Therefore, the purpose of this study was to examine the factor structures of two dimensions of the FUNDES-Adult. We expected that the factor structures of performance and capability dimensions of the FUNDES-Adult, version 7, would be different.

## Methods

This secondary data analysis study was part of a larger national survey conducted in Taiwan by the DEDS team (7). The present study was approved by the Research Ethics Committee of the Hualien Tzu Chi Hospital, Buddhist Tzu Chi Medical Foundation (IRB104-04-A;IRB107-46-B) and Joint Institutional Review Board, Taipei Medical University (TMU-JIRB), Taiwan. The de-identified data were retrieved from the database of the Taiwan Databank of Persons with Disability (TDPD) that included 144,850 adult-times who received the DEDS assessment in 201 authorized hospitals from November 2013 to January 2015 (6, 11, 15, 22, 23).

Certified testers associated with authorized hospitals in Taiwan administered the FUNDES-Adult, version 7, by interviewing the applicant's caregivers or him/herself (24). The certified testers were professionals licensed as physical therapists, occupational therapists, speech therapists, social workers, clinical psychologists, counseling psychologists, nurses, audiologists, special educators, and vocational evaluators. To ensure the number and quality of FUNDES testers, training programs were funded by the Taiwan central government with recruitment of all these licensed professionals, especially those in the DEDS hospitals. The training programs for certified testers covered the procedures of the DEDS and regulations (30 min), introduction to ICF and ICF-CY (30 min), introduction to assessment instruments [FUNDES-Adult (60 min), FUNDES-Child (40 min)], practice of assessment instruments (200 min), and the web-based platform for entry and storage of data (30 min). At the end of each training course, a paper-and-pencil test was administered to certify the attending professionals (24). By the end of 2014, there were about 7,700 certified testers in Taiwan (12). Names, identification (ID) number and other related information of all certified FUNDES testers are kept in the FUNDES tester personnel dataset for manpower quality control in the DEDS (12).

### Participants

To examine the factor structures of the FUNDES-Adult, version 7, the information of the FUNDES-Adult, version 7, in the TDPD (22) has been retrieved. To reduce the bias, data with missing items or “not applicable” items that were higher than 30% in one of the domains (i.e., Domains 1~6) of the FUNDES-Adult were excluded. After data cleaning, the data of 88,124 adults left for factor analyses. Based on the consideration of age, disability severity, disability type, and place of residence, we used multi-step probability proportional to size (PPS) sampling to obtain an exploratory factor analysis (EFA) sample of 8,730 people (about 10%), and from the remaining 79,394 people, 500 people were sampled in the same way for confirmatory factor analysis (CFA). There are no significant differences in age, sex, severity, disability type, and place of residence between samples and the whole group (*p* > 0.05). The EFA is mostly used when developing or compiling scales to understand which indicators should be selected or deleted, and which dimensions are under a construct. The CFA is mostly used after the development of a scale to check whether specific indicators fall under the expected dimensions of the theory. The main purpose is theoretical verification. The best sample size being used in CFA is 250∽500 persons (25).

Individuals with information in the TDPD were assessed *via* face-to-face interview (to applicants themselves or to caregivers of the applicants) and direct tested by physicians and a certified tester in the authorized hospitals. The databank included a record of demographic characteristics (including personal factors), the individual's body function and body structures, AP functioning, and some environmental factors.

The disability severity of one person with disability was determined in the medical examination stage of the DEDS (6, 7). Relevant ICF body function/structure categories for specific diagnoses were coded by physicians trained in using a 0–4-point qualifier (no problem = 0, mild = 1, moderate = 2, severe = 3, and profound = 4). A final summative severity level was determined based on decision rules for combining levels of severity among the individual body function/structure codes (26). There were nine types of disability in the DEDS system, and the first eight types are based on the eight body–function and body–structure chapters of the ICF. The nineth type is the rare disease group.

### Measurement

The FUNDES-Adult, version 7, has been developed in year of 2014, and used for training programs and part of national evaluation for determining the disability eligibility qualification (11). The team also examined the psychometric properties of internal consistency, test-retest, content validity, concurrent validity, and construct validity of the FUNDES-Adult, version 7 (5, 13, 14). The FUNDES-Adult, version 7 has 94 items, including 72 AP items with performance and capability dimensions of the first six domains, eight environmental items in Domain 7 (Do7, Environmental attributes) and 14 motor AP items in Domain 8 (Do8, Motor action). The first six domains are Cognition (Do1, item number, *n* = 6^*^2), Mobility (Do2, *n* = 5^*^2), Self-care (Do3, *n* = 4^*^2), Getting alone (Do4, *n* = 5^*^2), Life activities (Do5, *n* = 8^*^2), and Participation (Do6, *n* = 8^*^2). In Domain 5 (Life activities), there are two subdomains: Household activities (Do5–1, *n* = 4) and Work or school activities (Do5–2, *n* = 4). Do7 (Environmental attributes) includes items to measure the perceived environmental barriers (12). In Do8 (*n* = 7^*^2), each item has independence and capacity dimensions. For items with performance dimension, the question would be “because of your health condition in the past 30 days, how much difficulty did you have in … activities,” and it refers to the extent of restriction on participation in daily life with existed assistance. For items with capabilities dimension, the question would be “how much difficulty did you have if without assistive technology and without others' assistance,” and it refers to the extent of restriction on daily participation without assistance. In Do8, the item with independence dimension was the degree of other's help with existed assistive technology by interview, and the capacity item was rated after direct test by certified testers (27). In this study, only AP items of Domain 1∽6 and motor independence items of Domain 8 by interview were chosen for factor structure examination.

The Do1 Cognition domain, item D1.1 to item D1.6, is designed to assess cognitive and communication activities, including concentrating, remembering, problem solving, learning, and communicating; Do2 Mobility, item D2.1 to item D2.5, to assess mobility activities such as standing, moving around inside the home, getting out of the home and walking long distances; Do3 Self-care, item D3.1 to item D3.4, for assessing hygiene, dressing, eating and staying alone; Do4 Getting along, item D4.1 to item D4.5, for assessing difficulties of interactions with other people; Do5-1 Household activities, item D5.1 to item D5.4, for assessing difficulty with day-to-day household activities, and Do5-2, item D5.5 to item D5.8, for assessing difficulty with activities related to work or school; Do6 Participation, item D6.1 to item D6.8, for assessing difficulty of community activities, barriers and hindrances in the world around the respondent, and problems with other issues, such as maintaining personal dignity. Then, Do8 Motor action, independent dimension of item D8.1 to item D8.7 were rated by interview to answer dependence extent in basic motor activities in daily living, such as sit-to-stand, walking, picking up objects, buttoning, and tying a knot with existed assistive devices.

The possible scores to each item of Domain 1∽6 are 0: no difficulty, 1: mild difficulty, 2: moderate difficulty, 3: severe difficulty, and 4: extreme difficulty, and items with independent dimension of Domain 8 are 0: independence, 1: supervision or reminding; 2: mild assistance; 3: moderate assistance; and 4: full assistance. The AP scoring methods were following the 6 frames of reference for answering questions in the WHODAS 2.0 Manual (8).

The WHODAS 2.0 has been translated into 47 languages and dialects and is suitable for assessing health status and disability in a variety of settings and populations (28). Psychometric properties of the WHODAS 2.0 and/or the FUNDES-Adult have been evaluated for a number of clinical conditions, including, but not limited to, the following health conditions: musculoskeletal diseases (29–32), chronic diseases (31, 33–36), psychiatric conditions (29, 31, 33, 37–47), cancer (33, 48, 49), hearing impairment (50–52), visual impairment (50), stroke (10, 31, 33, 53–58), Parkinson's disease (59–62), spinal conditions (18, 63–65), traumatic brain injury (63, 66, 67), multiple sclerosis (68), persons with disabilities (22, 23, 35, 69, 70), older patients discharged from emergency departments (71) and general population (8, 72). Psychometric properties for WHODAS 2.0 36-item and/or FUNDES-Adult have generally been found adequate. For example, the internal consistencies at the domain and summary levels of the WHODAS 2.0 ranged from 0.59~0.99 (8), excellent internal consistency in all languages (alpha > 0.90) (28), and that of both performance and capability dimensions of the FUNDES-Adult ranged 0.90~0.99 (13, 18). The test–retest reliability of the WHOAS 2.0 had an intra-class coefficient of 0.69~0.89 at item level; 0.93~0.96 at domain level; and 0.98 at overall level (8). The test–retest reliability of Domain 1~6 and Domain 8 of FUNDES-Adult was 0.40~0.99 (ICCs) for 30 adults with spinal cord injuries (12).

To our knowledge, only few studies examine the factor structures of different language versions of the WHODAS 2.0 36 items (8, 31, 43, 58, 70). The lack of consistency with original developers of WHODAS 2.0 may indicate the needs of future investigation of the factor structure. Besides, the factor structures of the FUNDES-Adult have been examined in two studies (13, 18), also demonstrated some inconsistent findings. The study of Chiu et al. was based on persons with spinal cord injuries (18), and the CFA study of Yen et al. was only based on the 6-domain assumption (13). The differences between performance dimension and capability dimension of the FUNDES-Adult could be used to understand the possible impacts of environmental factors (5). Therefore, the factor structure of both dimensions of the FUNDES-Adult, version 7, were examined in this study. We used one sample to obtained the factor structure by the EFA first and then used another sample to check the good fitness of that factor structure by the CFA.

### Data Reduction and Statistical Method

For factor analysis, we used the performance scores of 36 items of Domain 1∽6 of the FUNDES-Adult, version 7^t^, that translated and derived from 36 items of the WHODAS 2.0 to examine the factor structure of the performance dimension. Using the capability scores of Domain 1∽6 and independence scores of the Domain 8 that modified for disability eligibility evaluation to examine the factor structure of the capability dimension. Due to culture reasons, most scores of the item D3.4 (“Staying by yourself for a few days?”) and item D4.5 (sexual activities) were 9 (not applicable). These two items were deleted before the factor analysis. Therefore, there were 34 items for factor analyses of the performance dimension. The team also found that item D8.4 (stand up from chair) was almost the same as item D2.2 (Standing up from sitting). The item D8.4 item was then also deleted before the factor analysis. Totally, 40 items were used for factor analyses of the capability dimension.

Factor analysis is a statistical method used to describe variability among observed, correlated variables in terms of a potentially lower number of unobserved variables called factors. Users of factor analysis believe that it helps to deal with data sets where there are large numbers of observed variables that are thought to reflect a smaller number of underlying/latent variables.

Statistical analyses and the EFA were performed using SPSS 20.0 (IBM SPSS Statistics, Chicago, IL, USA, 2016). Since most observed item distributions violated the normality assumptions and were inter-correlated, we used the iterative principal axis factoring followed by oblique promax rotation (73). Factorability of items was examined by the Bartlett test (α was set at 0.05) and the Kaiser–Meyer–Olkin (KMO) measure of sampling adequacy. For EFA, a value of KMO >0.6 is tolerable (74) and of >0.8 is good fit (75). The number of factors was decided by multiple methods including eigenvalues > 1 and scree tests. Factor loadings ≥ 0.3 were considered salient loadings (73). The extracted latent factors were then named based on conceptual interpretation of the items.

We used correlation matrix to understand the correlations between each pair of factors and used structural equation modeling (SEM) of SPSS AMOS V.20 to analyze the CFA. If there were significant correlations at the factor level and overall level, the two-level hierarchical structure (second-order confirmatory factor) will be presented (76–78). To assess model fit, the fit indices with their cutoff criteria [goodness-of-fit index (GFI) ≥ 0.95, normed fit index (NFI) ≥ 0.95, comparative fit index (CFI) ≥0.95, Tucker–Lewis index (TLI) ≥ 0.95, and root mean square error of approximation (RMSEA) <0.06 was excellent and <0.08 was acceptable] were used (79, 80).

## Results

The population was 88,124 adults with disabilities. Their mean age was 53.02 ± 16.84 years old, ranged from 18 to 110 years. The EFA of psychometric properties of the FUNDES-Adult, version 7, was examined based on 8,730 adults with disabilities (aged 52.98 ± 16.81 years), and the other 500 people with disability (aged 54.30 ± 16.81 years) were used for CFA (Table 1). There were no significant differences in gender, age, disability type, and severity of disability between the EFA and CFA samples (*p* > 0.05) (Table 1). For disability type of our samples, most of them were with impairment of “mental functions and structures of the nervous system.” We compared the samples between those with and without that particular disability type, respectively, there was no significant differences in every type (*p* > 0.05).

**Table 1:** Demographic data of the study sample.

**Characteristics**	**All**	**Sample of EFA**	**Sample of CFA**	**EFA vs. CFA sample**,
	***n =* 88,124**	***n =* 8730**	***n =* 500**	**Statistics (p-value)**
**Age**				
Mean ± SD^a^	53.0 ± 16.84	52.9 ± 16.81	54.3 ± 16.81	−0.025 (0.98)
Sex, *n* (%)^b^				
Male,	51481 (58.4)	5082 (58.2)	308 (61.1)	2.233 (0.14)
Female	36643 (41.6)	3648 (41.8)	192 (38.4)	
**Disability type**, ***n*** (%)^b^^*^				
Chapter 1.	41314 (46.9)	4024 (46.1)	213 (42.6)	2.325 (0.13)
Chapter 2.	12375 (14.0)	1220 (14.0)	76 (15.2)	0.588 (0.44)
Chapter 3	2069 (2.3)	218 (2.5)	10 (2.0)	0.485 (0.49)
Chapter 4	8181 (9.3)	814 (9.3)	52 (10.4)	0.644 (0.42)
Chapter 5	2912 (3.3)	255 (2.9)	17 (3.4)	0.379 (0.54)
Chapter 6	5960 (6.8)	585 (6.7)	38 (7.6)	0.607 (0.44)
Chapter 7	25964 (29.5)	2668 (30.6)	161 (32.2)	0.598 (0.44)
Chapter 8	514 (0.6)	54 (0.6)	5 (1.0)	1.08 (0.30)
Others	142 (0.2)	19 (0.2)	1 (0.2)	0.007 (0.93)
**Disability severity**, ***n*** **(%)^b^**				
Mild	37108 (42.1)	3735 (42.8)	209 (41.8)	3.113 (0.37)
Moderate	28324 (32.1)	2807 (32.2)	153 (30.6)	
Severe	12895 (14.6)	1251 (14.3)	72 (14.4)	
Profound	9797 (11.1)	937 (10.7)	66 (13.2)	

* *Disability type was identified by individual's primary diagnosis and based on b/s Chapter; each individual may have more than one type of diagnosis. Chapter 1: mental functions/structures of the nervous system; Chapter 2: sensory functions (b2)/the eye, ear, and related structures (s2); Chapter 3: voice and speech functions/structures; Chapter 4: functions/ structures of the cardiovascular, haematological, immunological, and respiratory systems; Chapter 5: functions/structures of the digestive, metabolic, and endocrine systems; Chapter 6: genitourinary and reproductive functions/structures; Chapter 7: neuromusculoskeletal and movement-related functions/structures; Chapter 8: functions/structures of the skin and related structure*.

a *Independent t-test*.

b *Chi-square test*.

*EFA, exploratory factor analysis; CFA, confirmatory factor analysis*.

### Exploratory Factor Analysis

At first, we used EFA to analyze 34 items of performance dimension and 40 items of capability dimension separately, and to delete the items with <0.3 factor loading value base on analyses results and in expert meetings.

The EFA yielded five-factor FUNDES-Adult structures with a variance of 73.5% (Table 2) and of 75.9% (Table 3) for the performance and capability dimensions, respectively. For the performance dimension, the first factor included 10 items (D1.1–D1.6 and D4.1–D4.4) and named as performance of learning and interaction, the second included 8 items (D2.1–D2.5, and D3.1–D3.3) and named as performance of mobility and self-care, the third included 4 items (D5.1–D5.4) and named as performance of housework, the fourth included 8 items (D6.1 to D6.8) and named as impact of health on participation performance, and the last domain included 4 items (D5.5–D5.8) and named as performance in work/school. The factors correlation matrix in Table 2 showed moderate correlations across all factors (*r* = 0.622∽0.677). The KMO value is 0.968 (*p* < 0.001). In this study, we calculated each performance factor score of each participant as the mean of the item values. For the five performance factors, the mean scores were 1.42 ± 1.508 (Factor 1, performance of learning and interaction); 0.99± 1.300 (Factor 2, performance of mobility and self-care); 1.78 ± 1.566 (Factor 3, performance of housework); 1.63 ± 1.349 (Factor 4, impact of health on participation performance); 3.27 ± 1.418 (Factor 5, performance in work/school), and with Cronbach-α of 0.96, 0.95, 0.99, 0.91, and 0.99, respectively.

**Table 2:** Factor loading of the performance dimension of the FUNDES-Adult by exploratory factor analysis (*n* = 8730).

**Items of performance dimension**	**Factor**				
	**1**	**2**	**3**	**4**	**5**
D1.6 Conversation	**0.928**	0.021	−0.072	−0.044	−0.018
D1.5 Understanding	**0.913**	0.141	−0.134	−0.123	−0.037
D1.3 Problem-solving	**0.865**	0.027	0.079	−0.123	0.031
D1.2 Remembering to do important things	**0.823**	0.070	−0.009	−0.061	−0.004
D4.1 Dealing with strangers	**0.796**	−0.022	−0.015	0.104	−0.008
D1.4 Learning a new work	**0.781**	−0.052	0.166	−0.034	0.055
D1.1 Concentration	**0.768**	0.090	0.034	−0.031	0.010
D4.2 Maintaining a friendship	**0.700**	−0.114	0.046	0.210	0.023
D4.3 Getting along with people close to	**0.677**	0.091	−0.074	0.175	−0.038
D4.4 Making new friends	**0.599**	−0.181	0.166	0.263	0.042
D3.2 Getting dressed	0.026	**0.945**	−0.063	−0.058	−0.011
D2.3 Moving around inside home	0.001	**0.932**	−0.007	−0.020	−0.012
D3.1 Washing whole body	0.034	**0.903**	−0.038	−0.046	−0.003
D2.2 standing up from sitting	0.056	**0.845**	0.028	−0.027	−0.007
D2.4 Getting out of home	−0.011	**0.797**	0.079	0.024	0.022
D3.3 Eating	0.175	**0.776**	−0.118	−0.017	−0.021
D2.1 Standing for long periods	−0.062	**0.529**	0.222	0.143	0.052
D2.5 Walking a long distance	−0.136	**0.526**	0.270	0.168	0.062
D5.2 Do important household tasks well	0.023	0.015	**0.990**	−0.055	−0.016
D5.3 Do all needed household work	−0.003	0.044	**0.984**	−0.050	−0.021
D5.4 Household work performed as quickly as needed	−0.007	−0.014	**0.981**	−0.020	−0.001
D5.1 Household responsibilities	0.043	0.024	**0.961**	−0.067	−0.017
D6.5 Health affects one's emotion	−0.001	−0.070	−0.045	**0.878**	−0.032
D6.7 Health affects family	0.011	−0.055	−0.103	**0.849**	−0.009
D6.6 Health affects family finances	−0.083	−0.036	−0.076	**0.829**	0.067
D6.3 Others affects one's dignity	0.080	0.072	−0.067	**0.715**	−0.040
D6.8 Doing things for relaxation or pleasure	0.094	0.062	0.070	**0.642**	−0.017
D6.4 Health affects time consumption	−0.051	0.210	0.018	**0.580**	−0.007
D6.1 Joining in community activities	0.124	0.033	0.257	**0.472**	0.004
D6.2 Because of environmental barriers	0.058	0.325	0.039	**0.388**	−0.003
D5.6 Do important work/school tasks well	0.007	0.007	−0.017	−0.012	**1.000**
D5.7 Getting done all needed work	0.004	0.010	−0.017	−0.012	**1.000**
D5.5 Day-to-day work/school	0.004	0.004	−0.020	0.002	**0.985**
D5.8 Work performed as quickly as needed	−0.001	−0.010	0.005	0.011	**0.979**
Variance explained (Total =73.5%)	17.1%	16.2%	15.6%	15.6%	9.0%
**Correlation matrix**					
Factor 2	0.622				
Factor 3	0.629	0.667			
Factor 4	0.677	0.632	0.631		
Factor 5	0.359	0.330	0.449	0.398	

***Factor 1**, Performance of learning and interaction; **Factor 2**, Performance of mobility and self-care; **Factor 3**, Performance of housework; **Factor 4**, Impact of health on participation performance; **Factor 5**, Performance in work/school. FUNDES-Adult, Functioning Disability Evaluation Scale-Adult Version; Factors' loadings in bold indicate that they have been selected in a factor*.

**Table 3:** Factor loading of the capability dimension of the FUNDES-Adult by exploratory factor analysis (*n* = 8730).

	**Factor**				
	**1**	**2**	**3**	**4**	**5**
D8.7 Sit onto the chair	**0.963**	−0.031	−0.006	−0.015	−0.001
D8.6 walk for 3m and return	**0.932**	0.076	−0.082	−0.026	0.008
D8.2 Button up	**0.914**	−0.255	0.222	−0.049	0.040
D8.3 Tie something	**0.887**	−0.238	0.224	−0.058	0.046
D8.1 Picking up a pen or spoon	**0.828**	−0.383	0.293	−0.003	0.027
D2.2 standing up from sitting	**0.824**	0.226	−0.086	0.001	−0.037
D8.5 Bend down to pick something up	**0.808**	0.254	−0.149	−0.026	0.013
D2.3 Moving around inside home	**0.801**	0.269	−0.111	0.010	−0.040
D3.2 Getting dressed	**0.765**	0.217	0.016	−0.016	−0.016
D3.3 Eating	**0.732**	−0.073	0.216	0.049	−0.006
D3.1 Washing whole body	**0.694**	0.302	0.004	−0.018	−0.012
D2.4 Getting out of home	**0.564**	0.457	−0.092	0.027	−0.010
D2.1 Standing for long periods	**0.519**	0.466	−0.135	0.073	−0.015
D2.5 Walking a long distance	**0.481**	0.526	−0.187	0.085	0.004
D5.4 Household work performed as quickly as needed	0.049	**0.842**	0.114	−0.042	0.043
D5.2 Do important household tasks well	0.086	**0.839**	0.142	−0.079	0.027
D5.3 Do all needed household work	0.115	**0.827**	0.117	−0.068	0.020
D5.1 Household responsibilities	0.093	**0.821**	0.150	−0.085	0.025
D6.1 Joining in community activities	0.009	**0.493**	0.180	0.265	−0.019
D1.6 Conversation	0.007	−0.015	**0.889**	−0.031	−0.023
D1.5 Understanding	0.113	−0.136	**0.857**	−0.041	−0.031
D1.3 Problem-solving	−0.006	0.188	**0.812**	−0.110	0.022
D4.1 Dealing with strangers	0.005	0.035	**0.779**	0.065	−0.010
D1.4 Learning a new work	−0.060	0.276	**0.771**	−0.084	0.026
D1.2 Remembering to do important things	0.007	0.111	**0.764**	−0.012	−0.007
D1.1 Concentration	0.105	0.062	**0.721**	−0.007	0.004
D4.2 Maintaining a friendship	−0.089	0.188	**0.697**	0.097	−0.002
D4.3 Getting along with people close to	0.102	−0.113	**0.668**	0.209	−0.019
D4.4 Making new friends	−0.165	0.289	**0.643**	0.126	0.012
D6.7 Health affects family	−0.067	−0.119	0.028	**0.874**	0.015
D6.5 Health affects one's emotion	−0.074	−0.026	0.042	**0.839**	−0.018
D6.6 Health affects family finances	−0.031	−0.090	−0.059	**0.835**	0.085
D6.3 Others affects one's dignity	0.078	−0.050	0.094	**0.700**	−0.024
D6.8 Doing things for relaxation or pleasure	0.082	0.186	0.116	**0.537**	−0.028
D6.4 Health affects time consumption	0.215	0.093	−0.013	**0.522**	−0.002
D6.2 Because of environmental barriers	0.274	0.169	0.049	**0.363**	−0.016
D5.7 Getting done all needed work	0.010	0.018	−0.011	0.006	**0.981**
D5.6 Do important work/school tasks well	0.006	0.017	−0.006	0.005	**0.981**
D5.5 Day-to-day work/school	0.009	0.011	−0.010	0.020	**0.960**
D5.8 Work performed as quickly as needed	−0.013	0.048	−0.014	0.021	**0.956**
Variance explained (Total variance = 75.9%)	19.0%	17.2%	16.1%	15.1%	8.4%
**Correlation matrix**					
Factor 2	0.668				
Factor 3	0.583	0.536			
Factor 4	0.576	0.660	0.636		
Factor 5	0.316	0.476	0.323	0.373	

***Factor 1**, Basic capability; **Factor 2**, Capability of housework; **Factor 3**, Capability of learning and interaction; **Factor 4**, Impact of health on participation capability; **Factor 5**, Capability in work/school. FUNDES-Adult, Functioning Disability Evaluation Scale-Adult Version; Factors' loadings in bold indicate that they have been selected in a factor*.

For the 40 items of the capacity dimension, the first domain included 14 items (D2.1–D2.5; D3.1–D3.3; D8.1–D8.3; and D8.5–D8.7), the second included 5 items (D5.1–D5.4; and D6.1), the third included 10 items (D1.1–D1.6; and D4.1–4.4), the fourth included 7 items (D6.2–D6.8), and the last domain included 4 items (D5.5–D5.8). The factors were named as “Basic capability,” “Capability of housework,” “Capability of learning and interaction,” “Impact of health on participation capability,” and “Capability in work/school.” The factors correlation matrix in Table 3 showed moderate correlations across all factors (*r* = 0.316∽0.668). The KMO value was 0.975 (*p* < 0.001). Each capability factor score of each participant was calculated by averaging the item scores that comprised. For the five capacity factors, the mean scores were 1.16 ± 1.508 (Factor 1, Basic capability); 2.17 ± 1.539 (Factor 2, Capability of housework); 1.53 ± 1.408 (Factor 3, Capability of learning and interaction); 1.68 ± 1.388 (Factor 4, Impact of health on participation capability); 3.32 ± 1.352 (Factor 5,Capability in work/school), and with Cronbach-α of 0.98, 0.97, 0.96,0.90, 0.90, and 0.99, respectively.

### Confirmatory Factor Analysis

**Figure 1:**
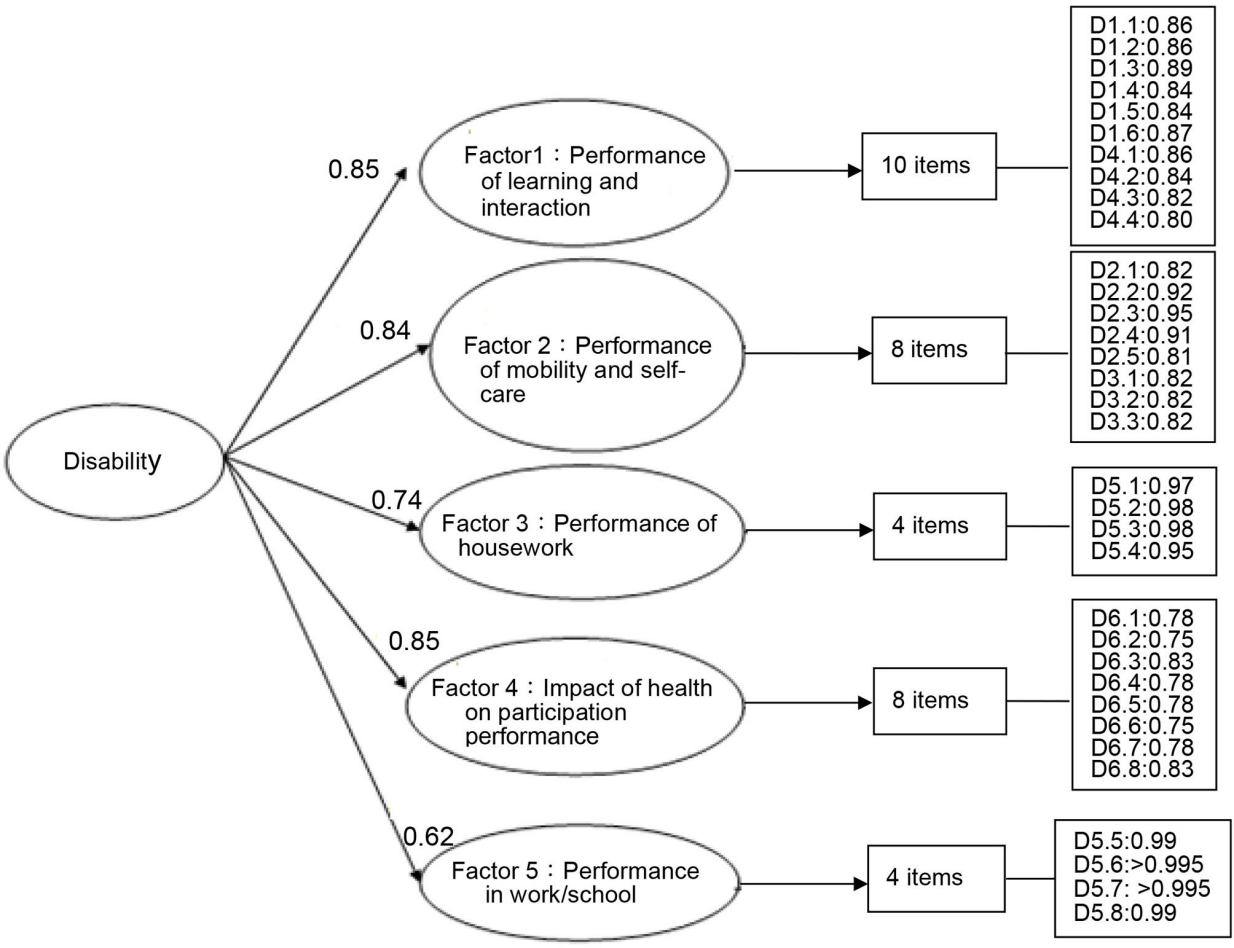
Factor structure of the performance dimension of the FUNDES-Adult, version 7, by the CFA. (*N* = 500, 34 items); Fit index (Bollen–Stine bootstrap *p*-correction estimated): TLI = 0.99, CFI = 0.99, NFI = 0.97, RMSEA = 0.028.

The ICC were ranged from 0.351 to 0.884 at the domain level and 0.655 (*p* < 0.05) at the overall level that meant that we must use multidimensional measurement to confirm the disability concept. The second-order CFA is one of the hierarchical measurement models. The factor loadings for the second-order CFA for the 34 items of performance dimension were from 0.62 to 0.85 (Figure 1) and the fit indices on this model were TLI = 0.99, CFI = 0.99, NFI = 0.97, and RMSEA = 0.028. All fit indices showed a good fit. All 34 items had factor loadings >0.75 on their corresponding factors, supporting the construct validity of the performance dimension.

**Figure 2:**
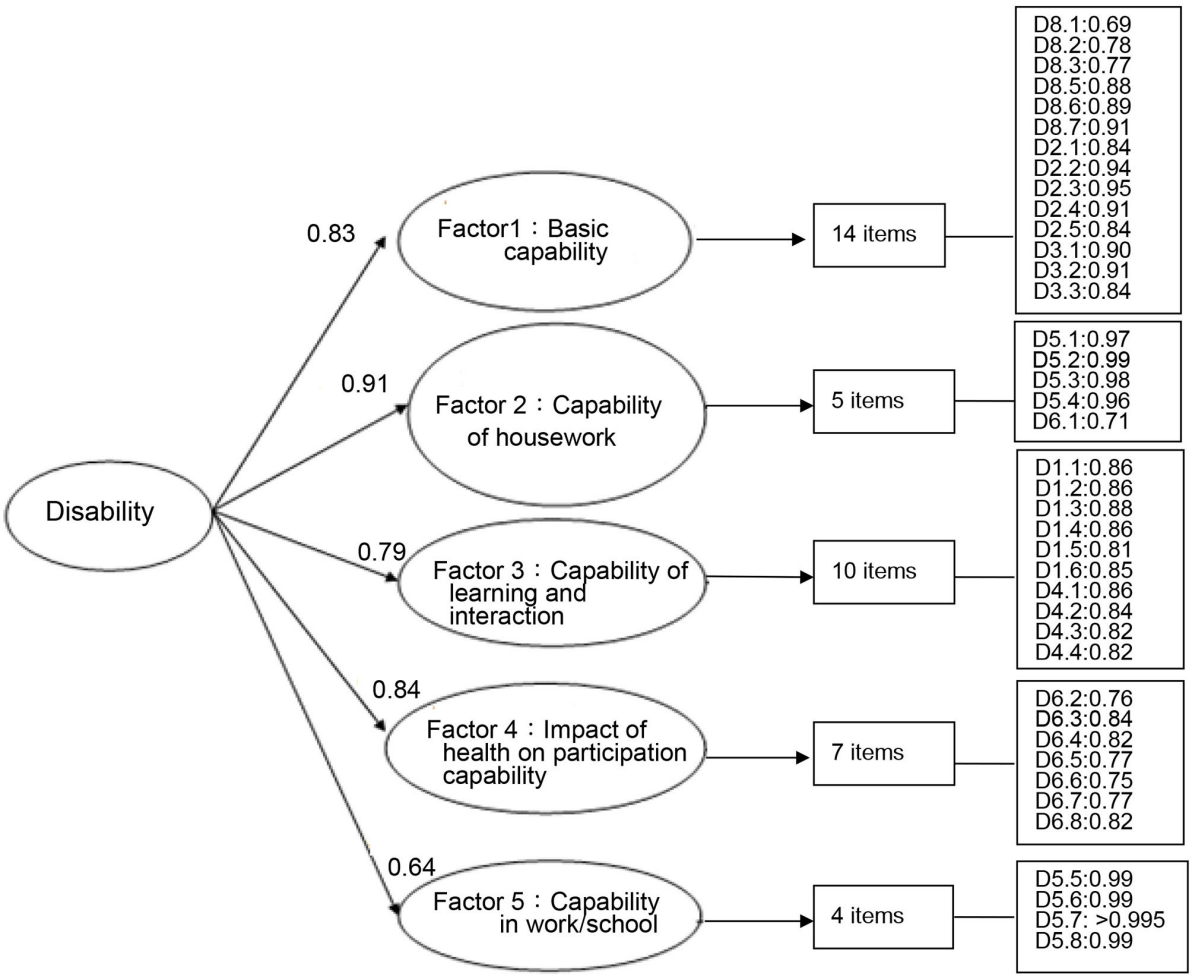
Factor structure of the capability dimension of the FUNDES-Adult, version 7, by the CFA (*N*= 500, 40 items); Fit indices (Bollen–Stine bootstrap *p*-correction estimated): TLI = 0.99, CFI = 0.99, NFI = 0.97, RMSEA = 0.087.

For the 40 items of capability dimension, the factor loadings for the second-order CFA were from 0.64 to 0.91(Figure 2). The fit indices of the model were TLI = 0.99, CFI = 0.99, NFI = 0.97, and RMSEA = 0.087, indicating acceptable to excellent model fit. All 40 items had factor loadings >0.69 on their corresponding factors, supporting the construct validity of the capability dimension.

## Discussion

Participation is one of the most significant outcomes of rehabilitation, social, and educational interventions (81). Using a large nationwide DEDS sample, the results of this study provided evidence of construct (structural) validity of the AP part of FUNDES-Adult by using both EFA and CFA for adults with disabilities and aged more than 18 years. The 5-factor structures of two dimensions of the FUNDES-Adult, version 7, are similar to our previous finding of FUNDES-Adult, version 5 (13). The items loading on each of the five factors of the performance dimension reflected the following domains: Performance of learning and interaction, Performance of mobility and self-care, Performance of housework, Impact of health on participation performance, and Performance in work/school; and that of capability dimensions are: Basic capability, Capability of housework, Capability of learning and interaction, Impact of health on participation capability, and Capability in work/school. This study also confirmed the second-order factor structure of the FUNDES-Adult. However, the items of each factor of two dimensions were different slightly and the names of the five factors were also different. The hypothesis of this study was partially supported. In Taiwan DEDS, the 5 domain scores and an overall disability score in two dimensions could be computed, each ranging from zero (indicating no perceived disability) to 100 (indicating maximum perceived disability). These disability scores are important for disability practices, research, and policies in the future in Taiwan.

Although Üstün et al. proposed the WHODAS 2.0 36-item version has a second-order factor structure in all cultures and populations tested (82) and it shows a mild correspondence with the theoretically six-domain structure. Such factor structure also found in the performance dimension of the FUNDES-Adult of persons with spinal cord injuries (18). However, the six-domain structure did not be confirmed by some previous studies (31, 58, 68, 70, 83). The possible reasons for somewhat different factor structures among the studies include differences in samples, influence of cultural and language differences, item numbers, and testing procedures between the initial WHODAS 2.0 and the Chinese FUNDES-Adult.

For example, the answers of “D3.4 Staying by himself or herself for a few days” in the WHODAS 2.0 were usually “not applicable” in Taiwan disability population. One of the reasons may be that most Chinese families are seldom core families and for those with disabilities are usually cared by other family members. They do not have the opportunities to be alone in our daily life in the past 30 days. These results are similar to the spinal cord injury studies which used the WHODAS 2.0 (65) or the FUNDES-Adult (18). Nielsen et al. (71) also found that aged persons discharged from emergency department in Denmark had more than 15% missing data in item D3.4. The item D4.5 “Sexual activities” was always missing due to the conservative culture and be refused to answer or the answer from the attitude of the respondent was not highly credible (71). The rating problem of the item D4.5 was also mentioned and suggested a cultural problem (68, 70). Therefore, we deleted this item in the FUNDES-Adult, version 10 (84).

For developing an ICF-based evaluation tool in Taiwan's DEDS, the FUNDES team has done some pilot studies (9, 85–87) and conducted a literature review for reliabilities and validities of the WHODAS 2.0 at the very beginning (87). The team then revised each item of the WHODAS 2.0 to be with performance dimension and capability dimension. Each item with performance dimension is almost just translated from the original English, with more detailed description of each item and adapting Chinese culture in the FUNDES Manual (27). For example, most male Taiwanese answered the item D5.1 (“Taking care of household responsibilities”) with “not applicable” (9), because male Taiwanese thought household activities were women's responsibilities. Therefore, the D5.1 question has been changed to” Taking care of household issues and family members.” A lot of examples are provided in the manual, including managing finances, car and house repairs, disciplining children, water flowers, etc. Some items that frequently confused testers were also revised based on the feedback of qualified testers. For example, item D1.4 (Learning a new task; for example, learning how to get to a new place?) was usually interpreted as testing the mobility problem due to the example sentence of “learning how to get to a new place”. We then revised the question of that item as “Learning a new task, for example, learning how to get to a new place and learning to use new daily living necessities and skills?”

For using the FUNDES-Adult as disability eligibility determination tools, the FUNDES team thought only the performance dimension is not enough. If performance dimension is the only criteria, one disabled person could possibly be deprived the disability qualification because of appropriate services and environmental support that leading to little restriction. We then add capability dimension to each item to measure the problem the applicants had without adaptive devices and others' assistance. Besides, to increase the better understanding of the capacity of the participants, the testers always started to test Do8 Motor action after demographic data collection. The process and results of the direct testing of the Do8 Motor action could help tester to deal with few applicants who responded most items with scores of 4 (profound) deliberately. The results of the Do8 could also to be used as later needs assessment related to mobility devices.

Using the EFA, Yen et al. found capability dimensions of the FUNDES-Adult, version 5, have five factors for 5,736 adults with disabilities (13); however, Chiu et al. found 6 factors in 521 adults with spinal cord injuries (18). As we mentioned before, differences of sample characteristics are possible reasons. Hence, we do not compare factor structures in different samples, disability group *vs*. spinal problems. We compared the factor structures between the FUNDES-Adult, version 5, and the FUNDES-Adult, version 7, further. It was found that the names of the five factors were the same, and only one item loaded in different factors. For FUNDES-Adult, version 5, the item D6.1 (How much of a problem did you have joining in community activities?) cross-loaded on three factors, Impact of health on participation capability factor (Factor loading = 0.577), Capability of learning and interaction factor (Factor loading = 0.359), and Capability of housework factor (Factor loading = 0.348) (13). The number of cross loading items might be due to the partial conceptual overlap between some aspects of the different domains of the WHODAS 2.0 (28). For FUNDES-Adult, version 7, the item D6.1 loaded only in the capability of housework factor (factor loading = 0.493). Therefore, the second-order 5-factor structures of two dimensions of the FUNDUS-Adult, version 7, was confirmed.

Besides the correlation matrix of the factor levels, we also calculated the omega reliability coefficients based on the formula proposed by McDonald (88, 89) and the estimated omega reliability coefficients were the average of the factor loadings in the Figures 1, 2 of this study (90). It showed that omega reliability coefficients were 0.78 and 0.80 for performance and capability dimension, respectively. Those are within the well-structured indicator of omega range of 0.75–0.83 (90).

Because most of the Do6 items of the FUNDESA-Adult, version 7, were loaded on a factor named as “Impact of health on participation performance. The modifications to the WHODAS 2.0 for the World Mental Health Surveys did not use original Do6 items (91). The FUNDES team then decided to add five new items and to keep the item D6.1 of the FUNDES, version 7, in the new Domain 6 (Societal participation) of the FUNDES-Adult, version 10. Then, we renamed the original Do6 as Do7 (Impact of health on participation) with seven items. The new five items of the Do6 (Societal participation) of the FUNDES-Adult 10th are derived from the Participation Measure−3 Domains, 4 Dimensions (PM-3D4D) (92, 93). The PM-3D4D is a 19-item measure that was developed on the basis of the conceptual model of participation (93). It is designed to evaluate participation in 3 domains—Productivity, Social, and Community—across 4 dimensions—Diversity, Frequency, Desire for change, and Difficulty. We selected and revised five items of Community domain of the PM-3D4D and Item D6.1 to form Do6 (Societal participation) of the FUNDES, version 10 (84).

Disability data is very important for the disability-inclusive development and for estimating disability prevalence (94). Guidebook published by the World Bank supports the implementation of the Washington Group Short Set (WG-SS) in multi-topic household surveys (95). The six questions of the WG-SS include seeing, hearing, walking, cognition, self-care, and communication. In the FUNDES-Adult, there were items related to walking, cognition, self-care, and communication functioning. Therefore, the FUNDES team have added seeing and hearing items in the FUNDES- Adult, version 10, and FUNDES-Child, version 10. The FUNDES, version 10, started to be used on 1 January 2022. Its psychometrics will be examined in the near future.

This study demonstrates the factor structure of the FUNDES-Adult, a modified assessment tool from the WHODAS 2.0, could be used as an ICF-based measure of AP successfully in the DEDS. The Taiwan's DEDS took the results of the FUNDES as references for the first stage of needs assessment to determine the supports related to parking space for persons with disabilities, transportation supports, and necessary accompany, and RehabBus services (a public transportation services to people with disabilities) (12, 85). The possibility of adjusting the disability grading by the FUNDES has been proposed. Right now, the disability grading or severity of the disability identification is decided mainly by the results of body structure and function assessment due to political and cultural reasons. The previous studies demonstrated there were significant correlations between body structure and function scores and FUNDES-Adult scores or WHODAS scores in each type of diagnosis or disability (30, 50, 51, 60). However, the FUNDES-Adult score of the extreme severe hearing impairment was lower than that of the mild stroke (22). That means that the disability grading based on body structure and function scores only is not fair. The impacts of combining body structure and function scores and FUNDES score on disability grading adjustment are under investigation. We expect the 5-factor structures of the FUNDES-Adult, version 7, could be used as references to adjust the disability grading when we collect enough data of the FUNDES, version 10, in the near future. The application of the FUNDES by clinicians or social welfare service providers to enhance the social participation and outcome evaluation tools for people with disabilities will be presented. However, the FUNDES-Adult requires additional testing and validation. For example, the WHODAS 2.0 shows as a valid instrument to assess functioning differences related to the clinical impact classification level in subjects with chronic obstructive pulmonary disease (36) and has the potential to become a patient-reported outcome measure (20). For persons with depression, most performance on the WHODAS 2.0 were improved after 2-year follow-up (40). We need to investigate the progress of other health conditions. Besides, the ICF-based collaborative problem-solving model could be used in the family-centered early intervention service process and the person-centered services (85, 96). We hope the main theme of ICF, enhancing the full participation of people with disabilities in society, could be reached through the application of the FUNDES.

## Data Availability Statement

The data analyzed in this study is subject to the following licenses/restrictions: The data set is an official data bank. Researchers can access the de-identification data through an application process. Requests to access these datasets should be directed to Head of the Department of Health and Welfare Services, Taiwan, through send the application letter to Taiwan Society of ICF, taiwansocietyicf@gmail.com and the website of the TSICF is http://www.icf.org.tw/.

## Ethics Statement

The studies involving human participants were reviewed and approved by the Research Ethics Committee of the Hualien Tzu Chi Hospital, Buddhist Tzu Chi Medical Foundation (IRB104-04-A;IRB107-46-B) and Joint Institutional Review Board Taipei Medical University (TMU- Joint Institutional Review Board), Taiwan. Written informed consent for participation was not required for this study in accordance with the national legislation and the institutional requirements.

## Author Contributions

C-FY and H-FL provided concept/idea/research design, data analysis, and writing. T-YC, W-CC, and T-HL provided data collection and data set maintenance as well as data analysis assistance. H-FL and T-HL provided project management. facilities/equipment, institutional liaisons, and administrative support. W-CC, B-SC, T-FW, and S-JL assisted testers training and data checking. All authors reviewed the manuscript before submission. All authors contributed to the article and approved the submitted version.

## Funding

The study was funded by Ministry of Health and Welfare/Taipei, Taiwan (M05F4145, M06F5054, M07F5223, M08F3147, M09F4089, and M1006158).

## Conflict of Interest

The authors declare that the research was conducted in the absence of any commercial or financial relationships that could be construed as a potential conflict of interest.

## Publisher's Note

All claims expressed in this article are solely those of the authors and do not necessarily represent those of their affiliated organizations, or those of the publisher, the editors and the reviewers. Any product that may be evaluated in this article, or claim that may be made by its manufacturer, is not guaranteed or endorsed by the publisher.
